# Vaginal lactobacilli inhibit growth and hyphae formation of *Candida albicans*

**DOI:** 10.1038/s41598-019-44579-4

**Published:** 2019-05-31

**Authors:** Sung Jae Jang, Kyeongju Lee, Bomi Kwon, Hyun Ju You, GwangPyo Ko

**Affiliations:** 1 Department of Environmental Health Sciences, Graduate School of Public Health, Seoul National University, Seoul, Republic of Korea; 2 Institute of Health and Environment, Seoul National University, Seoul, Republic of Korea; 3 KoBioLabs, Inc., Seoul, Republic of Korea; 4 Bio-MAX/N-Bio, Seoul National University, Seoul, Republic of Korea; 5 Center for Human and Environmental Microbiome, Institute of Health and Environment, Seoul National University, Seoul, Republic of Korea

**Keywords:** Antifungal agents, Applied microbiology

## Abstract

*Lactobacillus* species are the predominant vaginal microbiota found in healthy women of reproductive age and help to prevent pathogen infection by producing lactic acid, H_2_O_2_ and anti-microbial compounds. Identification of novel vaginal *Lactobacillus* isolates that exhibit efficient colonisation and secrete anti-*Candida* factors is a promising strategy to prevent vulvovaginal candidiasis. The azole antifungal agents used to treat vulvovaginal candidiasis elicit adverse effects such as allergic responses and exhibit drug interactions. *Candida* strains with resistance to antifungal treatments are often reported. In this study, we isolated *Lactobacillus* species from healthy Korean women and investigated their antifungal effects against *C*. *albicans in vitro* and *in vivo*. *Lactobacillus* conditioned supernatant (LCS) of *L*. *crispatus* and *L*. *fermentum* inhibited *C*. *albicans* growth *in vitro*. A *Lactobacillus*-derived compound, which was not affected by proteolytic enzyme digestion and heat inactivation, inhibited growth and hyphal induction of *C*. *albicans* after adjustment to neutral pH. Combination treatment with neutral LCSs of *L*. *crispatus* and *L*. *fermentum* effectively inhibited propagation of *C*. *albicans* in a murine *in vivo* model of vulvovaginal candidiasis.

## Introduction

*Lactobacillus* species are the predominant vaginal microbiota found in healthy women of reproductive age and inhibit pathogen growth by producing lactic acid, H_2_O_2_ and anti-microbial compounds^1–3^. Highly diverse *Lactobacillus*-non-dominant vaginal microbial communities are strongly correlated with genital inflammation, which negatively affects reproductive health and increases the risk of HIV infection^4,5^. On the other hand, *Lactobacillus gasseri*-dominant vaginal communities increase the clearance of human papillomavirus^6^. Furthermore, the abundance of *Gardnerella* or *Ureaplasma* species, which is related to the risk of preterm birth, is elevated in bacterial communities in which *Lactobacillus* species are lowly abundant^7^. These findings imply that *Lactobacillus* species in the vagina are important for preventing infection, clearing pathogenic microbes and modulating inflammation.

*Candida* species are the most common causes of fungal infection. Infections caused by *Candida* species affect 75% of women, and at least 6–9% of women experience recurrent vulvovaginal candidiasis. Among *Candida* species, *C*. *albicans* is the major cause of *Candida* infections in most countries. *C*. *albicans* was reported to account for 85–95% of yeast strains isolated from the vagina^8^. The risk factors for vulvovaginal candidiasis include hormonal changes, an immunocompromised state, pregnancy and antibiotic exposure^8^. Antifungal medications, such as clotrimazole and fluconazole, are used to treat *C*. *albicans* infections^9^. However, oral azoles exhibit drug interactions and can cause allergic responses. *Candida* strains with resistance to antifungal treatments are often reported^8^. Furthermore, oral azoles can potentially cause systemic toxicity. Topical azoles are safer; however, a few patients have experienced a burning sensation^10^.

The vaginal microbiome is unique and diverse and comprises various species. Identification of vaginal *Lactobacillus* species that produce an anti-*Candida* factor is a potential innovative strategy to prevent vulvovaginal candidiasis^8^. In this study, we isolated and characterised vaginal lactobacilli from healthy Korean women and investigated their anti-*Candida* activity and preventative effect on vulvovaginal candidiasis *in vitro* and *in vivo*.

## Results

### Isolation and characterisation of vaginal lactobacilli strains

**Figure 1:**
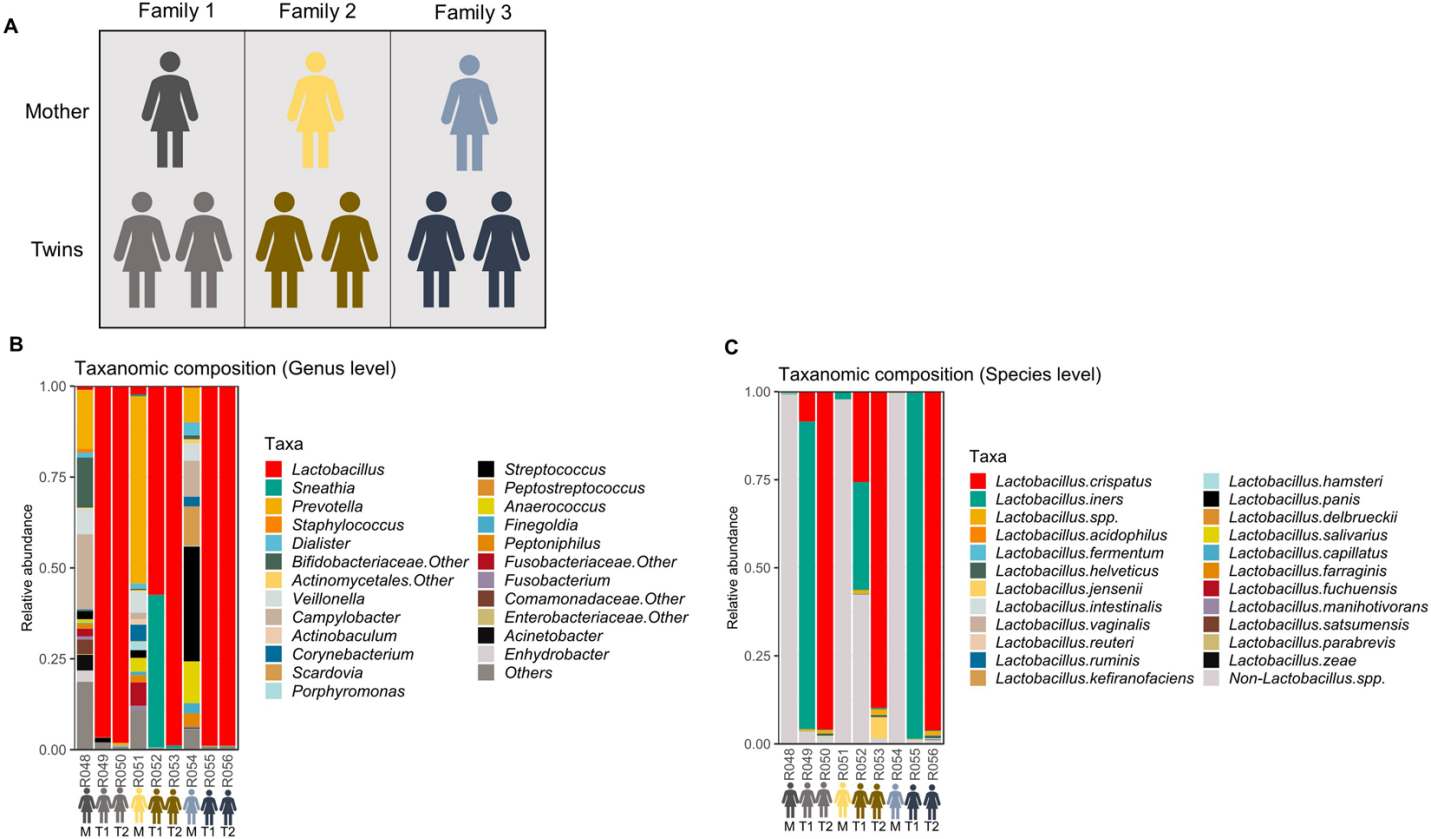
Vaginal microbiota composition of the nine study subjects. (**A**) Nine healthy Korean women were included as study subjects. (**B**,**C**) Composition of vaginal microbiota at the genus (**B**) and species (**C**) levels. Each family (1–3) comprised a pair of twins (T1 and T2) and their mother (M).

We investigated the compositional differences between the vaginal microbiota of healthy individuals using three sets of pre-menopausal Korean twins and their post-menopausal mothers (Fig. 1A). The composition of the vaginal microbiota differed between the subjects (Fig. 1B,C). In daughters, *Lactobacillus* species were the most abundant microbiota in the vaginal environment. The abundance of *Proteobacteria*, especially species belonging to the families *Enterobacteriaceae* and *Caulobacteraceae*, was much higher in mothers than in daughters. The microbiota was enriched with *Veillonella*, *Campylobacter*, *Scardovia* and *Streptococcus* at the genus level in mothers, but not in daughters (Fig. 1B). At the species level, *L*. *iners* and *L*. *crispatus* were the most abundant species in daughters, while the bacterial community was heterogenous in mothers. *L*. *iners* and *L*. *crispatus* were not dominant in mothers (Fig. 1C).

**Figure 2:**
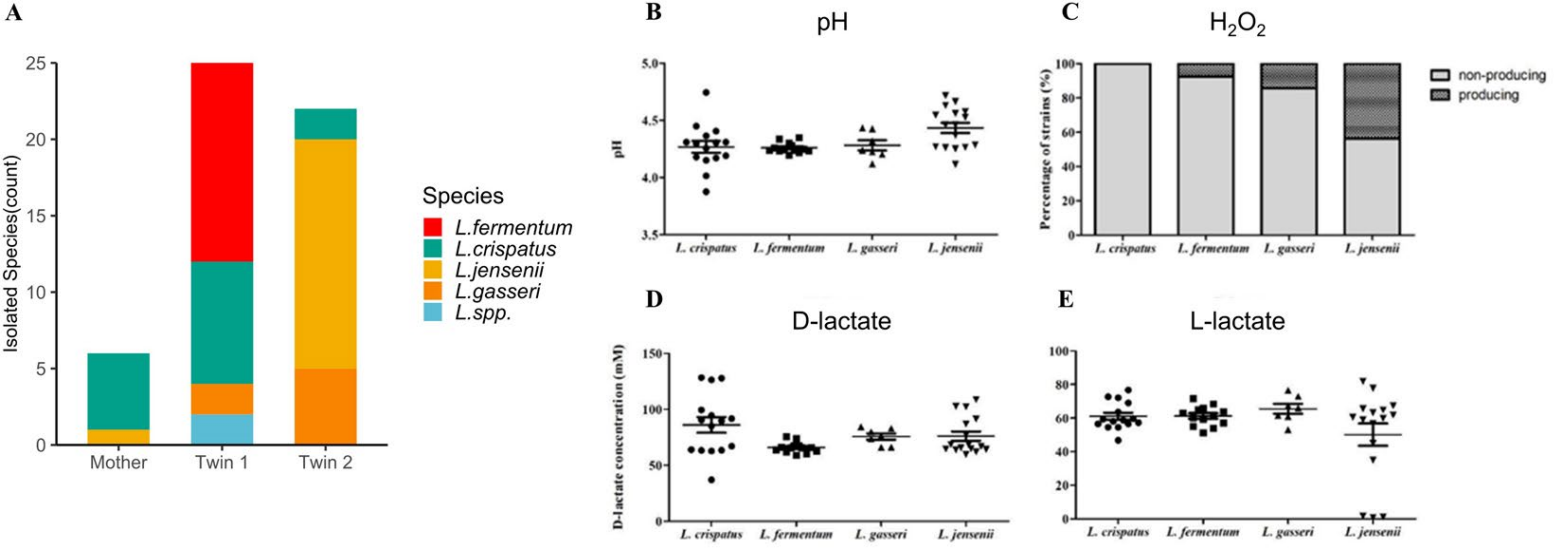
Characteristics of vaginal *Lactobacillus* isolates. (**A**) Fifty-one *Lactobacillus* strains were isolated from three healthy Korean women, namely, a pair of monozygotic twins (T1 and T2) and their mother. Colours in the bar graph denote the *Lactobacillus* species in each sample. (**B**) The pH of the LCS was measured using a benchtop pH meter. (**C**) H_2_O_2_ production was measured on TMB agar and expressed as the percentages of H_2_O_2_-producing and -non-producing strains in the stacked bar graph. (**D**,**E**) Concentrations of D-lactate (**D**) and L-lactate (**E**) produced by *Lactobacillus* species.

Fifty-one *Lactobacillus* isolates were obtained from vaginal specimens acquired from subjects belonging to Family 2. These isolates consisted of 15 *L*. *crispatus* strains, 13 *L*. *fermentum* strains, 7 *L*. *gasseri* strains and 16 *L*. *jensenii* strains (Fig. 2A and Supplementary Table S2). Six *Lactobacillus* strains were isolated from the mother (M), while 22 (T1) and 23 (T2) *Lactobacillus* strains were isolated from each twin daughter. Although subjects T1 and T2 were monozygotic twins with nearly identical DNA and subject M was their mother, they displayed unique distributions of *Lactobacillus* species in their vaginal isolates.

We evaluated the culture medium pH and H_2_O_2_ productivity of the 51 isolated *Lactobacillus* strains. The pH of *Lactobacillus* conditioned supernatant (LCS) was 3.88–4.75, showing that all the strains acidified the culture medium (Fig. 2B). H_2_O_2_ was not produced by most of the *Lactobacillus* strains; only 9 of the 51 *Lactobacillus* strains produced H_2_O_2_. H_2_O_2_ was produced by none of the *L*. *crispatus* strains, but by a high percentage of the *L*. *jensenii* strains (Fig. 2C). D-lactate was produced at a concentration of 37.1–128.3 mM by all the isolates. The *L*. *crispatus* SNUV220 strain produced the highest concentration of D-lactate (Fig. 2D,E). However, there was no remarkable difference among the species due to the wide concentration range. In general, the isolated *Lactobacillus* species produced more D-lactate than L-lactate.

### Antifungal effects and characterisation of LCSs

**Figure 3:**
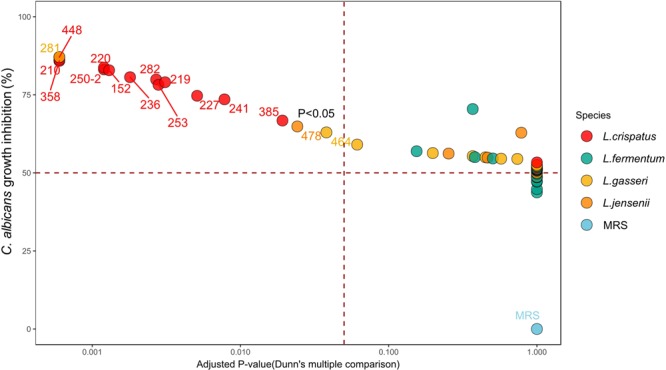
Anti-*Candida* activity of LCSs. Effects of pH-unadjusted acidic LCSs on *C*. *albicans* growth *in vitro*. Red, yellow, green, blue and orange represent *L*. *crispatus*, *L*. *fermentum*, *L*. *gasseri*, *L*. *jensenii* and MRS, respectively. Data represent the mean rate of *C*. *albicans* growth inhibition (%) in two independent experiments. The dotted line designates the significance level of p < 0.05.

The inhibitory effect of the 51 *Lactobacillus* strains on *C*. *albicans* growth was evaluated *in vitro*. We prepared LCSs of the lactobacilli isolates. The antifungal activities of the 51 LCSs were assessed and presented according to the rank of activity and adjusted p-values (Fig. 3). *C*. *albicans* growth was inhibited most by the LCS of the *L*. *gasseri* strain SNUV281, but was not markedly inhibited by those of the other *L*. *gasseri* strains. The LCSs of *L*. *crispatus* exhibited higher anti-*Candida* activity than those of the *L*. *fermentum*, *L*. *gasseri* and *L*. *jensenii* strains.

**Figure 4:**
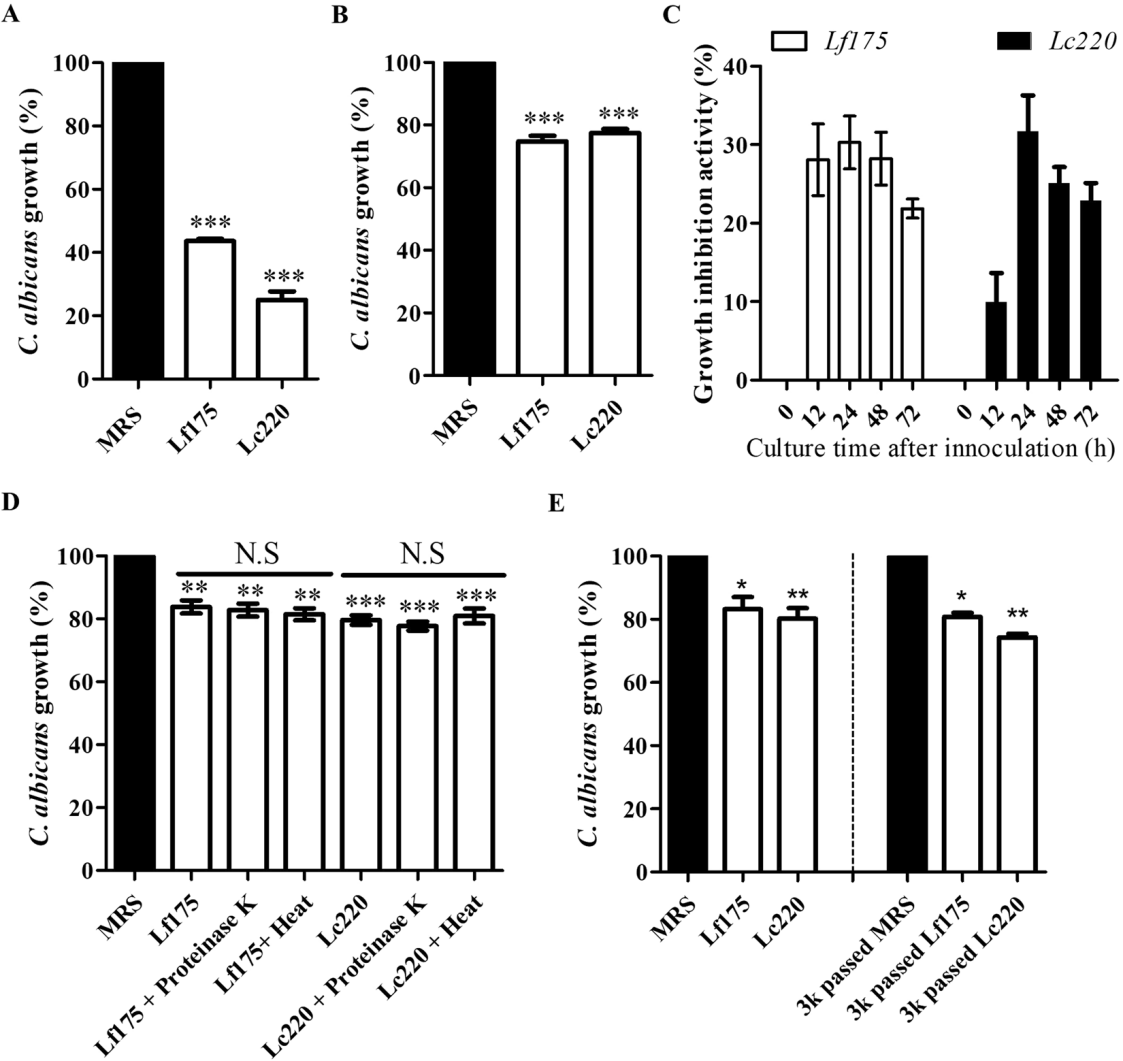
Inhibitory effects of LCSs from *L*. *crispatus* and *L*. *fermentum* on *C*. *albicans* growth. (**A**) Growth of *C*. *albicans* after treatment with acidic LCSs (pH 4.0–4.5) from Lf175 and Lc220. (**B**) Growth of *C*. *albicans* after treatment with neutral LCSs (pH 6.9) from Lf175 and Lc220. (**C**) Growth inhibitory effect of neutral LCSs prepared after incubation of lactobacilli for different durations. (**D**) Effect of protein digestion and heat inactivation on the anti-*Candida* activity of LCSs from Lf175 and Lc220. (**E**) Effect of size exclusion of LCSs by 3 kDa molecular weight filtration on anti-*Candida* activity. Lf175 and Lc220 represent *L*. *fermentum* SNUV175 and *L*. *crispatus* SNUV220, respectively. Data represent the mean and SEM of three independent experiments. Statistical significance was calculated using the Kruskal-Wallis test with Dunn’s multiple comparisons post hoc test. ***p < 0.005; **p < 0.001; *p < 0.05.

We selected the *L*. *crispatus* SNUV220 and *L*. *fermentum* SNUV175 strains for further analysis based on their anti-*Candida* growth activity and probiotic characteristics, such as resistance to gastric acidity, bile acid resistance and anti-microbial activity. We hypothesised that vaginal *Lactobacillus* strains secrete pH-independent antifungal compounds that inhibit *C*. *albicans* growth. The antifungal activity of the LCSs was evaluated. Treatment with pH-unadjusted LCSs of *L*. *fermentum* SNUV175 and *L*. *crispatus* SNUV220 decreased *C*. *albicans* growth by 43.7% ± 2.7% and 25.07% ± 11.3%, respectively (Fig. 4A). Both LCSs also inhibited *C*. *albicans* growth at neutral pH; LCSs of *L*. *fermentum* SNUV175 and *L*. *crispatus* SNUV220 decreased *C*. *albicans* growth by 74.81% ± 7.215% and 77.42% ± 5.633%, respectively (Fig. 4B). The inhibitory effect of acidity due to lactate production was also assessed. *C*. *albicans* growth was inhibited when the pH was decreased to 4.0 using HCl and to 4.5 using 500 mM L/D-lactate (Supplementary Fig. S1A,B). However, *C*. *albicans* growth was not affected by L-lactate, D-lactate and L/D-lactate (16.125–500 mM) after adjustment of the pH to 6.9 (Supplementary Fig. S2C).

Next, we investigated the time-course of the antifungal effects of the LCSs. The LCSs were harvested at 12, 24, 48 and 72 h after inoculation of *Lactobacillus*, and their anti-*Candida* activity was evaluated (Fig. 4C). The LCS of *L*. *fermentum* SNUV175 collected at 12–48 h exhibited strong antifungal activity, while the LCS of *L*. *crispatus* SNUV220 collected from 24 h displayed antifungal activity. The antifungal effect of the LCSs was not affected by heat inactivation and proteolytic enzyme treatment (Fig. 4D). Finally, the molecular weight of the antifungal molecule was estimated by 3 kDa molecular weight filtration (Fig. 4E). The LCSs of both *L*. *fermentum* SNUV175 and *L*. *crispatus* SNUV220 still elicited an antifungal effect after filtration. These results suggest that the inhibitory effect of the LCSs is attributable to a neutral small molecule (less than 3 kDa) that is not affected by heat and proteolytic enzymes.

### Inhibitory effect of neutral LCSs on hyphal growth

**Figure 5:**
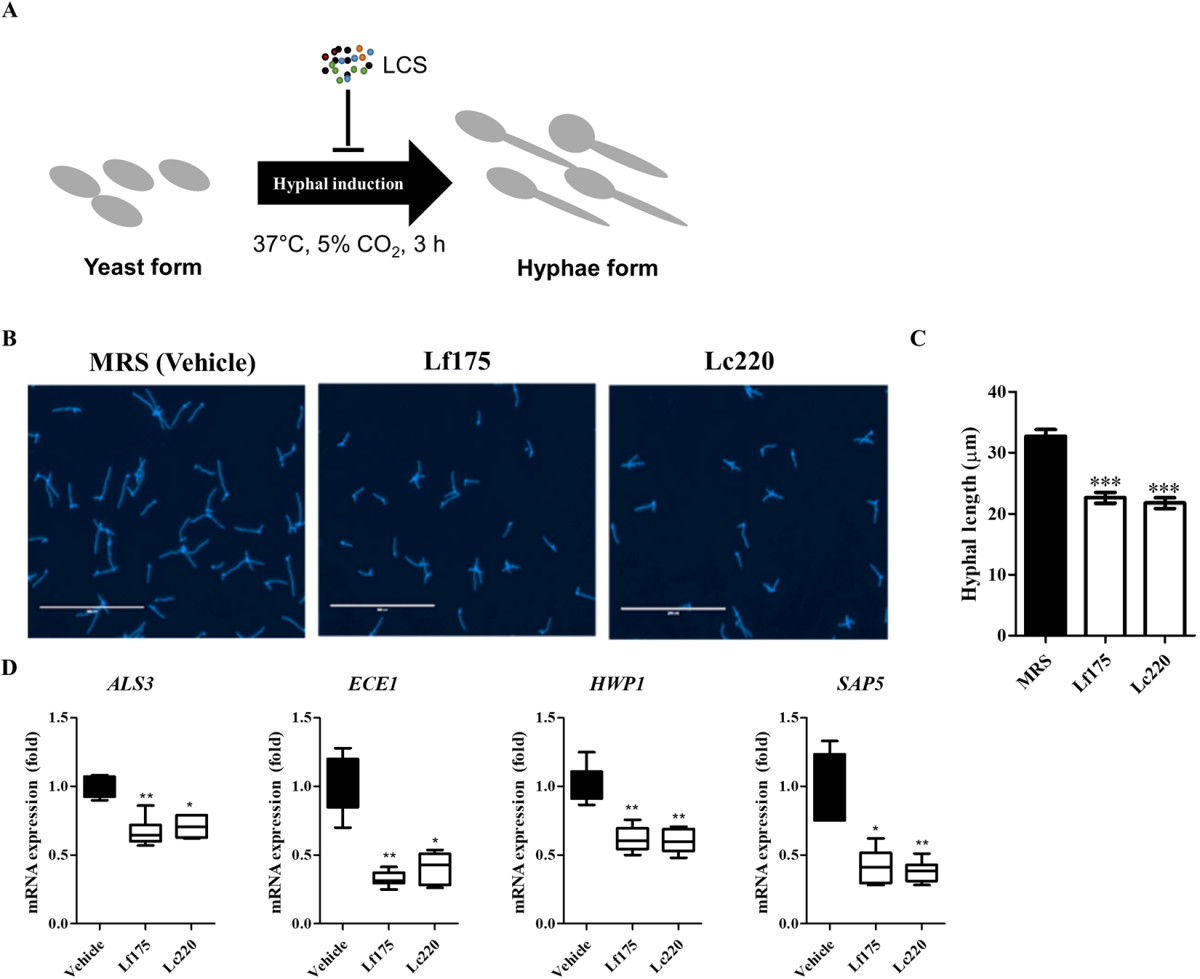
Inhibitory effects of LCSs from *L*. *crispatus* and *L*. *fermentum* on hyphal transition. (**A**) Schematic diagram of the hyphal growth assay. (**B**) Hyphae of *C*. *albicans* were treated with neutral LCSs of Lf175 and Lc220, and stained with Calcofluor white. Scale bar, 200 μm. (**C**) Hyphal length was manually measured. (**D**) qPCR analysis of the expression levels of *ALS3*, *ECE1*, *SAP5* and *HWP1*. Lf175 and Lc220 represent *L*. *fermentum* SNUV175 and *L*. *crispatus* SNUV220, respectively. Statistical significance was calculated using the Kruskal-Wallis test with Dunn’s multiple comparisons post hoc test. ***p < 0.005; **p < 0.001; *p < 0.05.

We investigated the effect of the LCSs from *L*. *fermentum* SNUV175 and *L*. *crispatus* SNUV220 on hyphal induction (Fig. 5A). Incubation with neutral LCSs significantly suppressed hyphal growth in comparison with the vehicle control (Fig. 5B,C). The pH-unadjusted acidic LCSs also inhibited hyphal growth well (Supplementary Fig. S1). We further investigated hypha-related gene expression by qPCR. Consistently, treatment with the neutral LCSs significantly downregulated expression of hypha-related genes, such as *ALS3*, *ECE1*, *SAP5* and *HWP1* (Fig. 5D).

### Anti-*Candida* activity of a mixture of neutral LCSs in a murine model of vulvovaginal candidiasis

**Figure 6:**
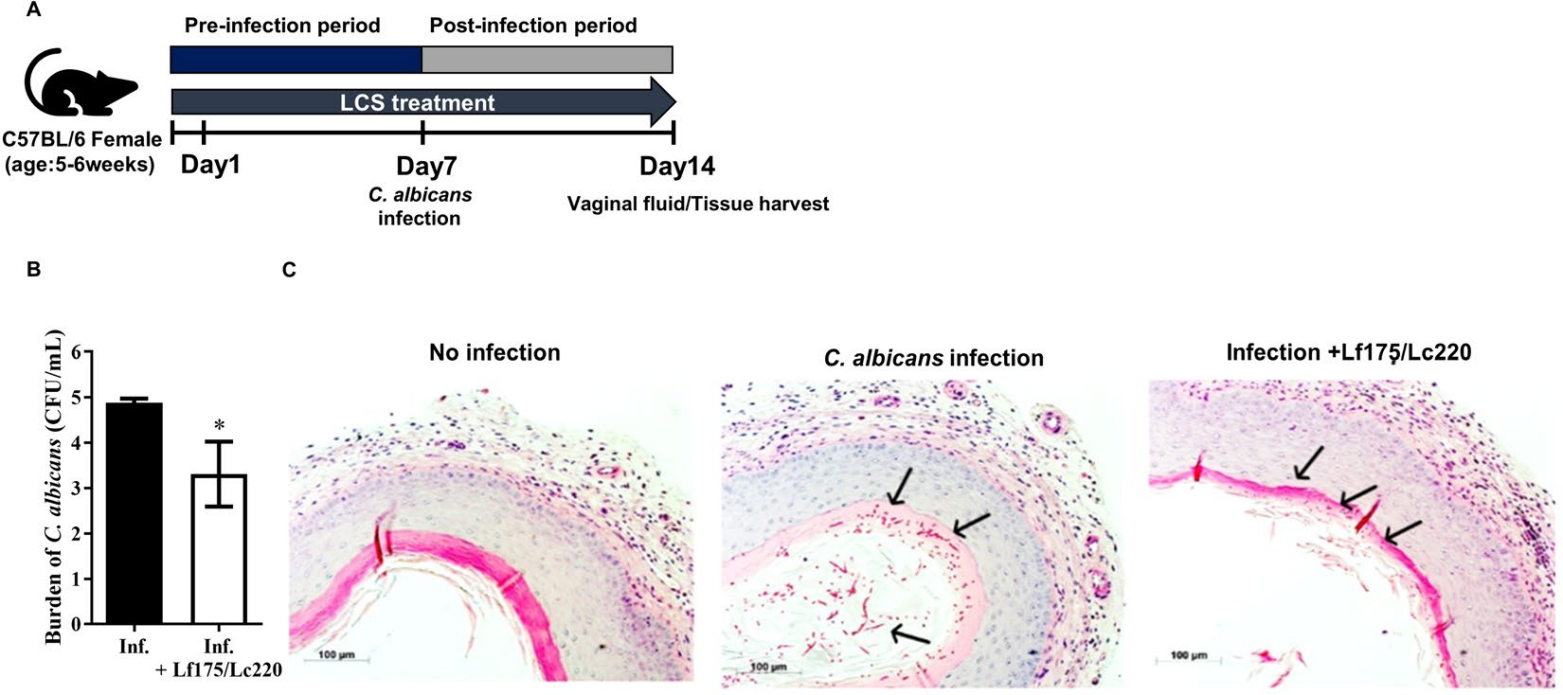
Effect of a mixture of LCSs on *C*. *albicans* growth in a murine model of vulvovaginal candidiasis. C57BL/6 mice were infected intravaginally with 5 × 10^6^ *C*. *albicans* cells. A neutral LCS mixture (20 μL) was administered each day for 7 days pre-infection and 7 days post-infection. (**A**) Schematic overview of the murine model of vulvovaginal candidiasis. (**B**) Colony forming units (CFUs) of *C*. *albicans* in vaginal fluid were counted on SDAC agar. Data represent the means and SEM. Inf, infection group (N = 6); Inf + Lf175/Lf220, group treated with a mixture of LCSs (N = 7). (**C**) Histological evaluation of vaginal infection of *C*. *albicans* by Periodic acid-Schiff staining. Scale bar, 100 μm. Black arrows indicate *C*. *albicans* in vaginal tissue. Lf175 and Lc220 represent *L*. *fermentum* SNUV175 and *L*. *crispatus* SNUV220, respectively. Statistical significance was calculated using the Mann-Whitney U test. ***p < 0.005; **p < 0.001; p < 0.05.

We investigated the anti-*Candida* effect of a mixture of neutral LCSs from *L*. *fermentum* SNUV175 and *L*. *crispatus* SNUV220 in a murine model of vulvovaginal candidiasis (Fig. 6A). The burden of *C*. *albicans* in the vagina was significantly decreased after treatment with this LCS mixture intravaginally for 2 weeks (Fig. 6B and Supplementary Fig. S3). Furthermore, histological evaluation revealed that *C*. *albicans* clearance was increased, consistent with the results obtained *in vitro* (Fig. 6C).

## Discussion

This study focused on isolation of vaginal lactobacilli that exhibit strong anti-*Candida* activity *in vitro* and *in vivo*. We first analysed the vaginal microbiota composition and lactobacilli species profiles of Korean women, and isolated 51 vaginal lactobacilli strains comprising four *Lactobacillus* species. We isolated four strains (*L*. *fermentum*, *L*. *crispatus*, *L*. *jensenii* and *L*. *gasseri*) in an unbiased manner. Their distribution was similar among the subjects. At the species level, the abundance of *L*. *iners* and *L*. *crispatus* was high in metagenome analysis. However, *L*. *iners* strains were not isolated. These findings were consistent among the subjects. The distribution of *Lactobacillus* species was similar in similar study^11^ but depending on the disease states and isolation methods, various species of bacteria can be isolated from vagina fluid^12^.

*C*. *albicans* growth was inhibited more by the majority of pH-unadjusted, acidic LCSs of *L*. *crispatus* than by those of other *Lactobacillus* species. The vaginal environment in humans is maintained at an acidic pH (4–4.5) via production of lactate by lactobacilli, and this is important to inhibit pathogen growth^13^. However, the acidic environment and dominance of lactobacilli are not sustained after menopause, and consequently post-menopausal women are more susceptible to diverse infections. The LCSs of *L*. *fermentum* strains exhibited potent anti-*Candida* activity when adjusted to neural pH and at their acidic naïve pH, suggesting that these strains produce antifungal molecules other than lactate^14^.

A recent study reported that the culture supernatant of *L*. *crispatus* at low pH suppresses growth and hyphal growth of *C*. *albicans in vitro*^15^. Other lactobacilli species, including *L*. *rhamnosus*, *L*. *reuteri* and a mixture thereof, were reported to be vaginal probiotics that inhibit pathogen growth^16–18^. *L*. *fermentum* elicits broad inhibitory effects on *Candida* species^19^. In this study, *L*. *fermentum* and *L*. *crispatus* isolates inhibited *C*. *albicans* growth at neutral pH effectively *in vitro* assay and *in vivo* model. Moreover, the LCSs inhibited *C*. *albicans* morphogenesis to develop hyphae, which is a virulent form that causes vaginal inflammation^20,21^. Hyphal growth was reported to be important for virulence of *C*. *albicans*^22–26^. The candidalysin peptide (*ECE1*) is only secreted by the hyphal form of *C*. *albicans* and induces lysis of mammalian cell membranes, inflammation and pathogenesis of vulvovaginal candidiasis^27,28^. Expression of hyphal-related genes, such as *ALS3*, *ECE1*, *SAP5* and *HWP1*, was significantly downregulated in the LCS-treated groups^29,30^. *ALS3* contributes to invasion of cells and cellular damage^21^. *HWP1* encodes a cell wall mannose protein required for hyphal formation and adhesion to epithelial cells^31^. *SAP5* encodes a member of the secreted aspartic protease family, which is important for the pathogenesis of candidiasis^32^. Expression of virulence genes is related to the pathogenesis of *C*. *albicans* infection. Therefore, the decrease in hypha-related genes is expected to alleviate cellular damage and inflammation induced by *C*. *albicans*.

Rodent models of vaginal infection do not completely mimic the vaginal environment of humans^33^. However, many murine models of vulvovaginal candidiasis have been reported^28,34,35^. A mixture of LCSs at neutral pH inhibited *C*. *albicans* growth in a murine vulvovaginal candidiasis model. This suggests that *Lactobacillus* strains can prevent candidiasis in the non-acidic vaginal environment by inhibiting growth and morphogenesis of *Candida*. We further investigated the biochemical traits of the anti-*Candida* compound produced by *L*. *crispatus* and *L*. *fermentum*. Antifungal activity at neutral pH was not affected by heat inactivation and proteolytic enzyme treatment. The compound responsible was less than 3 kDa in size and was produced by lactobacilli after the stationary phase. An antifungal protein was previously reported to inhibit hyphal growth^15^; however, the antifungal compounds reported in that previous study and the current study appear to differ. Further research is required to extract the anti-*Candida* compound from LCS and to determine its molecular structure by bioassay-guided fractionation and comparative metabolite analysis.

In summary, vaginal *Lactobacillus* strains isolated from healthy women produced a small molecule with anti-*Candida* activity in addition to lactate and inhibited growth and hyphal morphogenesis of *C*. *albicans*. We used three models to validate the anti-*Candida* effect of lactobacilli, namely, an *in vitro* assay, a hyphal growth assay and an *in vivo* model of vulvovaginal candidiasis. Our results suggest that resistance to vulvovaginal candidiasis is increased when *L*. *crispatus and L*. *fermentum* is the dominant bacterial community in the vaginal environment. The antifungal compound produced by vaginal *Lactobacillus* may reduce the burden of *C*. *albicans* infection. Future studies are required to elucidate the mechanism by which growth and hyphal transition of *C*. *albicans* are suppressed at the molecular level.

## Methods

### Isolation of vaginal *Lactobacillus* strains from healthy Korean subjects

Study subjects were recruited from the Healthy Twin Study as part of the Korean Genome Epidemiology Study^36^. All participants provided written informed consent to participate in this study. Samples were collected from three pairs of monozygotic twins and their mothers who underwent Papanicolaou smear tests at Samsung Medical Center (Seoul, Korea). The age of the nine subjects ranged from 25 to 79 years (Supplementary Table S3). Among the subjects, mothers (M) were post-menopausal and twins (T1 and T2) were pre-menopausal. None of the participants had any history of cervicovaginal disease and any genetic/metabolic diseases. Cervicovaginal samples were collected from the mid-vaginal wall during a speculum examination by clinicians using an ESwab (Copan Diagnostics Inc., Murrieta, CA, USA)^37^. The swabs were immediately stored in modified Liquid Amies solution, placed on ice and transported to the laboratory for microbiome analysis and lactobacilli isolation^38^. *Lactobacillus* species were isolated on to Rogosa medium(Oxoid Ltd, Basingstoke, Hampshire, UK), Brain-Heart infusion medium (Becton, Dickinson and Company, Baltimore, MD, USA, Columbia medium(Oxoid Ltd, Basingstoke, Hampshire, UK) and Chocolate medium (Oxoid Ltd, Basingstoke, Hampshire, UK)The study protocol was approved by the Korea Centers for Disease Control and the Institutional Review Board (IRB) of Samsung Medical Center (IRB No. 144-2011-07-11). All experiments were performed in accordance with relevant guidelines and regulations.

### DNA extraction and 16S rRNA sequencing analysis

Total genomic DNA was extracted from vaginal swabs using a PowerSoil^®^ DNA Isolation Kit (MO BIO Laboratories, Inc., Carlsbad, CA, USA) according to the manufacturer’s instructions with minor modifications^39^. Extracted nucleic acids were stored at −80 °C until use. The V4 region of the 16S rRNA gene was amplified using Illumina adaptor universal primers (515F/806R) and the 16S rRNA Amplification Protocol from the Earth Microbiome Project^40^. The PCR amplicon was purified using an UltraClean^®^ PCR Clean-Up Kit (MO BIO Laboratory, Inc., Carlsbad, CA, USA) and quantified using a Quant-iT PicoGreen dsDNA Assay Kit (Life Technologies, Carlsbad, CA, USA). The samples were pooled and sequenced on a MiSeq platform with a 2 × 300 bp reagent kit (Illumina, San Diego, CA, USA)^41^. The generated reads underwent quality filtering and trimming using the FASTX-Toolkit. Sequence data were analysed using Quantitative Insights Into Microbial Ecology (QIIME) 1.5.0 (http://qiime.sourceforge.net)^42^. Open-reference operational taxonomic unit picking was performed at the 97% sequence similarity level with reference to the Greengene database (gg_12_10). Taxonomic composition analysis was performed to compare taxonomic abundance between the groups.

### Microbial strains and culture conditions

Fifty-one *Lactobacillus* strains were isolated from vaginal swabs of three healthy Korean women using Rogosa agar. Lactobacilli were routinely grown in De Man, Rogosa and Sharpe (MRS) medium (Becton, Dickinson and Company, Baltimore, MD, USA) containing 0.05% L-cysteine hydrochloride anaerobically at 37 °C. *C*. *albicans* ATCC^®^ MYA-4788 was purchased from the American Type Culture Collection. *C*. *albicans* was routinely grown in Yeast Extract-Peptone-Dextrose (YPD) medium (10 g/L of yeast extract, 20 g/L of peptone and 20 g/L of D-glucose) in aerobic conditions at 30 °C for 18 h. All bacterial and fungal stocks were stored at −80 °C in the presence of 17% glycerol as a cryoprotectant.

### Measurement of lactate and H_2_O_2_ production by *Lactobacillus* species and the pH of LCSs

All isolates were identified by Sanger sequencing of the 16S rRNA target region (27F/1492R). Lactic acid production by *Lactobacillus* strains was investigated using EnzyChrom L- and D-lactate Assay kits (BioAssay Systems, Hayward, CA, USA). *Lactobacillus* strains isolated from the vagina were cultured in MRS broth medium and subsequently filter-sterilised. The lactate concentration in the LCS was measured in accordance with the manufacturer’s instructions. The acidity of the LCS was measured using a benchtop pH meter (Thermo Fisher Scientific, Waltham, MA, USA). The ability of *Lactobacillus* strains to produce H_2_O_2_ was evaluated as described by Rabe and Hillier^43^ with minor modifications. All strains were cultured on MRS agar plates containing 25 mg of 3,3′,5,5′tetramethylbenzidine (Sigma-Aldrich, St. Louis, MO, USA), 0.5 mg of hemin and 0.05 µg of vitamin K (Sigma-Aldrich, St. Louis, MO, USA). The plates were incubated anaerobically at 37 °C for 48 h and then exposed to air for 30 min to check for a blue colour change.

### Preparation of LCSs

Lactobacilli were grown in MRS broth anaerobically at 37 °C for 48 h and then removed by centrifugation at 4,000 × g for 10 min at 4 °C. The pH of the LCS was measured and adjusted to neutral with 5 N sodium hydroxide. Thereafter, the LCS was passed through a 0.22 µm nitrocellulose filter (Advantec Manufacturing Inc, New Berlin, WI, USA). The filtrate was stored at −20 °C before use.

### *C*. *albicans* growth inhibition assay

*C*. *albicans* was cultured in YPD broth for 18 h at 30 °C in a shaking incubator at 200 rpm, washed twice with 10 mM phosphate-buffered saline (PBS) at pH 7.4 and adjusted to a density of 2 × 10^6^ cells/mL using PBS. Thereafter, 100 µL of LCS, 100 µL of YPD broth and 50 µL of *C*. *albicans* suspension were added to each well of a 96-well culture plate (SPL Life Sciences Co., Ltd., Pocheonsi, Gyeonggido, Korea). MRS broth was used as a control for LCS. The culture plate was incubated aerobically at 30 °C for 24 h. Growth of *C*. *albicans* was measured spectrophotometrically at 600 nm.

### Hyphal growth inhibition assay

Hyphal growth was assessed based on hyphal length analysis^44^. *C*. *albicans* was cultured in YPD broth at 30 °C for 18 h in a shaking incubator at 200 rpm, washed twice with PBS and adjusted to a density of approximately 5 × 10^5^ cells/mL using serum-free Roswell Park Memorial Institute (RPMI) 1640 medium. For hyphal growth analysis, 100 µL of *C*. *albicans* cell suspension, 700 µL of fresh RPMI 1640 medium and 200 µL of LCS (20% v/v) were mixed in a 24-well culture plate. The plate was incubated at 37 °C in 5% CO_2_ for 3 h. Thereafter, the medium was removed, and *C*. *albicans* cells were fixed in 4% paraformaldehyde and stained with Calcofluor white (Sigma-Aldrich, St. Louis, MO, USA). Hyphae were imaged using an EVOS FL cell imaging system (Thermo Fisher Scientific, Waltham, MA, USA). Hyphal length was measured using ImageJ software (National Institutes of Health, Bethesda, MD, USA). All hyphal branches were included in length measurements.

### RNA extraction and quantification of gene expression

For gene expression analysis, *C*. *albicans* was cultured in YPD medium at 30 °C for 18 h in a shaking incubator at 200 rpm, washed twice with PBS and resuspended in serum-free RPMI 1640 medium. Subsequently, 1 mL of *C*. *albicans* (1 × 10^7^ cells), 7 mL of fresh RPMI 1640 medium and 2 mL of LCS (20% v/v) were mixed in a 100 mm^2^ culture dish, and then incubated at 37 °C in 5% CO_2_ for 3 h. Thereafter, the medium was removed, and *C*. *albicans* was rinsed with cold PBS, collected using a cell scraper, washed with 1 mL of cold PBS and centrifuged at 3,000 × g for 2 min at 4 °C. The supernatant was removed, and the cell pellet was stored at −80 °C prior to RNA extraction. RNA was extracted using a Yeastar™ RNA Kit (Zymo Research, Irvine, CA, USA) according to the manufacturer’s instructions. cDNA was synthesised using 500 µg of RNA as the template and a High Capacity RNA-to-cDNA Kit (Applied Biosystems, Foster City, CA, USA) according to the manufacturer’s instructions. cDNA samples were used for quantitative PCR with KAPA SYBR^®^ FAST qPCR Kit Master Mix (Kapa Biosystems, Wilmington, MA, USA). Amplification was performed using a Rotor Gene-Q system (Qiagen, Germantown, MD, USA). The PCR primer sequences are shown in Supplementary Table S1. The *Δ*Ct value was calculated using the *ACT1* gene as an endogenous control and normalised against the MRS control to calculate the 2^−(∆∆Ct)^ value for statistical analysis.

### Murine model of vulvovaginal candidiasis

Experiments using the murine model of vulvovaginal candidiasis were performed in accordance with the Guidelines for the Care and Use of Laboratory Animals issued by the Institutional Animal Care and Use Committee of Seoul National University, Faculty of Science (SNU-170801-7). The model of vaginal *C*. *albicans* infection was generated as described by Yano *et al*.^35^ and Borghi *et al*.^34^ with minor modifications. Briefly, 0.5 mg of β-estradiol 17-valerate (Sigma-Aldrich, St. Louis, MO, USA) diluted in 100 µL of sesame oil was administered intraperitoneally at 48 h before infection to maintain pseudo-estrous conditions and was administered weekly thereafter. LCS (20 µL) was administered intravaginally each day for 7 days pre-infection and 7 days post-infection with anaesthetics. *C*. *albicans* (5 × 10^6^ cells/mL) prepared in 10 µL of PBS containing 1% low-melting agarose (Lonza, Basel, Switzerland) was administered intravaginally. At 7 days post-infection, all mice were anesthetised, and vaginal fluid was collected to evaluate the burden of fungal infection. Vaginal tissues were fixed with 4% paraformaldehyde for 24 h and embedded in paraffin. Paraffin blocks were cut into 6 µm thick sections and mounted on slides. Vaginal mucosa and *C*. *albicans* were stained using Periodic acid-Schiff.

### Proteolytic enzyme treatment and heat inactivation

The LCS was adjusted to neutral pH with 5 N sodium hydroxide, treated with 4 U/mL proteinase K (V3021; Promega Corporation, Madison, WI, USA) for 1 h at 55 °C and heat-inactivated at 95 °C for 30 min.

### Size exclusion filtration

Size exclusion filtration was performed using 3 K Microsep™ Advance Centrifugal Devices (OD003C33; Pall Corporation, Washington, NY, USA). The filtrate was subjected to the *C*. *albicans* growth inhibition assay.

### Statistical analysis

All data were analysed with Prism 5 (GraphPad Software, San Diego, CA, USA) and R software 3.5.1. Two groups were compared using the Mann-Whitney U test. More than two groups were compared using the Kruskal-Wallis test with Dunn’s multiple comparisons test. In all graphs, data were presented as the mean and standard error of the mean (SEM). Statistical significance was denoted as follows: *p-value < 0.05, **p-value < 0.01 and ***p-value < 0.001.

## Supplementary information

Vaginal lactobacilli inhibit growth and hyphae formation of <i>Candida albicans</i>

## Data Availability

The datasets generated during and/or analysed during the current study are available from the corresponding author on reasonable request.
