# Supplementary material for: Vaginal lactobacilli inhibit growth and hyphae formation of Candida albicans
Source: Sci Rep. 2019 May 31;9:8121. doi: 10.1038/s41598-019-44579-4 (PMC6544633; doi:10.1038/s41598-019-44579-4)
Supplement: Supplementary file 1 — Vaginal lactobacilli inhibit growth and hyphae formation of Candida albicans [file 41598_2019_44579_MOESM1_ESM.pdf]

# Vaginal lactobacilli inhibit growth and hyphae formation of *Candida albicans*

Sung Jae Jang[1,6], Kyeongju Lee[1,6], Bomi Kwon[1], Hyun Ju You[1,2,*] and GwangPyo Ko[1,3,4,5,*]

[1]Department of Environmental Health Sciences, Graduate School of Public Health, Seoul National University, Seoul, Republic of Korea

[2]Institute of Health and Environment, Seoul National University, Seoul, Republic of Korea

[3]KoBioLabs, Inc., Seoul, Republic of Korea

[4]Bio-MAX/N-Bio, Seoul National University, Seoul, Republic of Korea

[5]Center for Human and Environmental Microbiome, Institute of Health and Environment, Seoul National University, Seoul, Republic of Korea

[6]Both authors equally contributed to this work as co-1st authors.

**Correspondence:**

GwangPyo Ko, Ph.D., gko@snu.ac.kr

Hyun Ju You, Ph.D., dhlover1@snu.ac.kr

## Supplementary Tables

Table S1. Primers used for qPCR analysi

| Gene | Sequence |
|---|---|
| *ACT1*-F[a] | TCAGACCAGCTGATTTAGGTTTG |
| *ACT1*-R[b] | GTGAACAATGGATGGACCAG |
| *ALS3*-R | CCTGAAATTGACATGTAGCA |
| *ALS3*-F | CTAATGCTGCTACGTATAATT |
| *ECE1*-F | GCTGGTATCATTGCTGATAT |
| *ECE1*-R | TTCGATGGATTGTTGAACAC |
| *HWP1*-F | TGGTGCTATTACTATTCCGG |
| *HWP1*-R | CAATAATAGCAGCACCGAAG |
| *SAP5*-F | CAGAATTTCCCGTCGATGAGA |
| *SAP5*-R | CATTGTGCAAAGTAACTGCAACAG |

[a]F represents a forward primer

[b]R represents a reverse primer

Table S2. Lactobacillus strains isolated from vaginal specimens of healthy women

| No. | Species | Strains | Source | Confirmed_ID |
|---|---|---|---|---|
| 1 | *L.crispatus* | 152 | Twin 1 | Lactobacillus crispatus strain G10 16S ribosomal RNA gene, partial sequence |
| 2 | *L.crispatus* | 210 | Mother | Lactobacillus crispatus strain G10 16S ribosomal RNA gene, partial sequence |
| 3 | *L.crispatus* | 219 | Mother | Lactobacillus crispatus ST1 complete genome, strain ST1 |
| 4 | *L.crispatus* | 220 | Mother | Lactobacillus crispatus strain A014 16S ribosomal RNA gene, partial sequence |
| 5 | *L.crispatus* | 227 | Twin 1 | Lactobacillus crispatus strain G10 16S ribosomal RNA gene, partial sequence |
| 6 | *L.crispatus* | 236 | Twin 1 | Lactobacillus crispatus strain G10 16S ribosomal RNA gene, partial sequence |
| 7 | *L.crispatus* | 241 | Twin 1 | Lactobacillus crispatus strain G10 16S ribosomal RNA gene, partial sequence |
| 8 | *L.crispatus* | 250 | Twin 1 | Lactobacillus crispatus strain G10 16S ribosomal RNA gene, partial sequence |
| 9 | *L.crispatus* | 253 | Twin 1 | Lactobacillus crispatus strain G10 16S ribosomal RNA gene, partial sequence |
| 10 | *L.crispatus* | 282 | Twin 2 | Lactobacillus crispatus strain A014 16S ribosomal RNA gene, partial sequence |
| 11 | *L.crispatus* | 358 | Twin 2 | Lactobacillus crispatus strain A014 16S ribosomal RNA gene, partial sequence |
| 12 | *L.crispatus* | 367 | Twin 1 | Lactobacillus crispatus ST1 strain ST1 16S ribosomal RNA, complete sequence |
| 13 | *L.crispatus* | 381 | Mother | Lactobacillus crispatus strain G4 16S ribosomal RNA gene, partial sequence |
| 14 | *L.crispatus* | 385 | Mother | Lactobacillus crispatus strain G10 16S ribosomal RNA gene, partial sequence |
| 15 | *L.crispatus* | 476 | Twin 1 | Lactobacillus crispatus ST1 strain ST1 16S ribosomal RNA, complete sequence |
| 16 | *L.fermentum* | 155 | Twin 1 | Lactobacillus fermentum strain IMAU32493 16S ribosomal RNA gene, partial sequence |
| 17 | *L.fermentum* | 157 | Twin 1 | Lactobacillus fermentum strain TY5 16S ribosomal RNA gene, partial sequence |
| 18 | *L.fermentum* | 168 | Twin 1 | Lactobacillus fermentum gene for 16S ribosomal RNA, partial sequence, strain: YS2 |
| 19 | *L.fermentum* | 175 | Twin 1 | Lactobacillus fermentum strain A045 16S ribosomal RNA gene, partial sequence |
| 20 | *L.fermentum* | 240 | Twin 1 | Lactobacillus fermentum strain TY5 16S ribosomal RNA gene, partial sequence |
| 21 | *L.fermentum* | 245 | Twin 1 | Lactobacillus fermentum strain A045 16S ribosomal RNA gene, partial sequence |
| 22 | *L.fermentum* | 257 | Twin 1 | Lactobacillus fermentum strain A045 16S ribosomal RNA gene, partial sequence |
| 23 | *L.fermentum* | 258 | Twin 1 | Lactobacillus fermentum strain A045 16S ribosomal RNA gene, partial sequence |
| 24 | *L.fermentum* | 259 | Twin 1 | Lactobacillus fermentum strain L4 16S ribosomal RNA gene, partial sequence |
| 25 | *L.fermentum* | 264 | Twin 1 | Lactobacillus fermentum strain CMUL54 16S ribosomal RNA gene, partial sequence |
| 26 | *L.fermentum* | 374 | Twin 1 | Lactobacillus fermentum gene for 16S ribosomal RNA, partial sequence, strain: S107D |
| 27 | *L.fermentum* | 430 | Twin 1 | Lactobacillus fermentum gene for 16S ribosomal RNA, partial sequence, strain: YS2 |
| 28 | *L.fermentum* | 444 | Twin 1 | Lactobacillus fermentum strain BCS30 16S ribosomal RNA gene, partial sequence |
| 29 | *L.gasseri* | 159 | Twin 1 | Lactobacillus gasseri strain BLB1b 16S ribosomal RNA gene, partial sequence |
| 30 | *L.gasseri* | 281 | Twin 2 | Lactobacillus jensenii strain A084 16S ribosomal RNA gene, partial sequence |
| 31 | *L.gasseri* | 431 | Twin 1 | Lactobacillus gasseri strain BLB1b 16S ribosomal RNA gene, partial sequence |
| 32 | *L.gasseri* | 457 | Twin 2 | Lactobacillus gasseri strain VEL 16S ribosomal RNA gene, partial sequence |
| 33 | *L.gasseri* | 462 | Twin 2 | Lactobacillus gasseri 16S ribosomal RNA gene, partial sequence |
| 34 | *L.gasseri* | 463 | Twin 2 | Lactobacillus gasseri strain BLB1b 16S ribosomal RNA gene, partial sequence |
| 35 | *L.gasseri* | 464 | Twin 2 | Lactobacillus gasseri strain BLB1b 16S ribosomal RNA gene, partial sequence |
| 36 | *L.jensenii* | 181 | Twin 2 | Lactobacillus jensenii strain A084 16S ribosomal RNA gene, partial sequence |
| 37 | *L.jensenii* | 184 | Twin 2 | Lactobacillus jensenii strain A084 16S ribosomal RNA gene, partial sequence |
| 38 | *L.jensenii* | 185 | Twin 2 | Lactobacillus jensenii strain A084 16S ribosomal RNA gene, partial sequence |
| 39 | *L.jensenii* | 190 | Twin 2 | Lactobacillus jensenii strain G16 16S ribosomal RNA gene, partial sequence |
| 40 | *L.jensenii* | 200 | Twin 2 | Lactobacillus jensenii strain G16 16S ribosomal RNA gene, partial sequence |
| 41 | *L.jensenii* | 226 | Mother | Lactobacillus jensenii strain A084 16S ribosomal RNA gene, partial sequence |
| 42 | *L.jensenii* | 287 | Twin 2 | Lactobacillus jensenii strain A084 16S ribosomal RNA gene, partial sequence |
| 43 | *L.jensenii* | 290 | Twin 2 | Lactobacillus jensenii strain A084 16S ribosomal RNA gene, partial sequence |
| 44 | *L.jensenii* | 291 | Twin 2 | Lactobacillus jensenii strain A084 16S ribosomal RNA gene, partial sequence |
| 45 | *L.jensenii* | 354 | Twin 2 | Lactobacillus jensenii strain A084 16S ribosomal RNA gene, partial sequence |
| 46 | *L.jensenii* | 360 | Twin 2 | Lactobacillus jensenii strain A084 16S ribosomal RNA gene, partial sequence |
| 47 | *L.jensenii* | 448 | Twin 2 | Lactobacillus jensenii partial 16S rRNA gene, strain Gasser 62G |
| 48 | *L.jensenii* | 465 | Twin 2 | Lactobacillus jensenii strain A084 16S ribosomal RNA gene, partial sequence |
| 49 | *L.jensenii* | 470 | Twin 2 | Lactobacillus jensenii strain A084 16S ribosomal RNA gene, partial sequence |
| 50 | *L.jensenii* | 478 | Twin 2 | Lactobacillus jensenii strain A084 16S ribosomal RNA gene, partial sequence |
| 51 | *L.jensenii* | 479 | Twin 2 | Lactobacillus jensenii strain G16 16S ribosomal RNA gene, partial sequence |
| 52 | *L.* spp. | 230 | Twin 1 | Uncultured Lactobacillales bacterium clone 4426 16S ribosomal RNA gene, partial sequence |
| 53 | *L.* spp. | 362 | Twin 1 | Lactobacillus sp. JCM 1035 gene for 16S rRNA, partial sequence, strain: JCM 1035 |

Yellow, green and blue denote isolates obtained from T1, T2 and their mother, respectively

Table S3. Study subjects

| SampleID | Family | Stage_of_Menopause | Age(years) |
|---|---|---|---|
| R048 | F1 | Post-menopause | 62 |
| R049 | F1 | Pre-menopause | 41 |
| R050 | F1 | Pre-menopause | 41 |
| R051 | F2 | Post-menopause | 71 |
| R052 | F2 | Pre-menopause | 44 |
| R053 | F2 | Pre-menopause | 44 |
| R055 | F3 | Post-menopause | 61 |
| R056 | F3 | Pre-menopause | 39 |
| R057 | F3 | Pre-menopause | 39 |

## Supplementary Figures

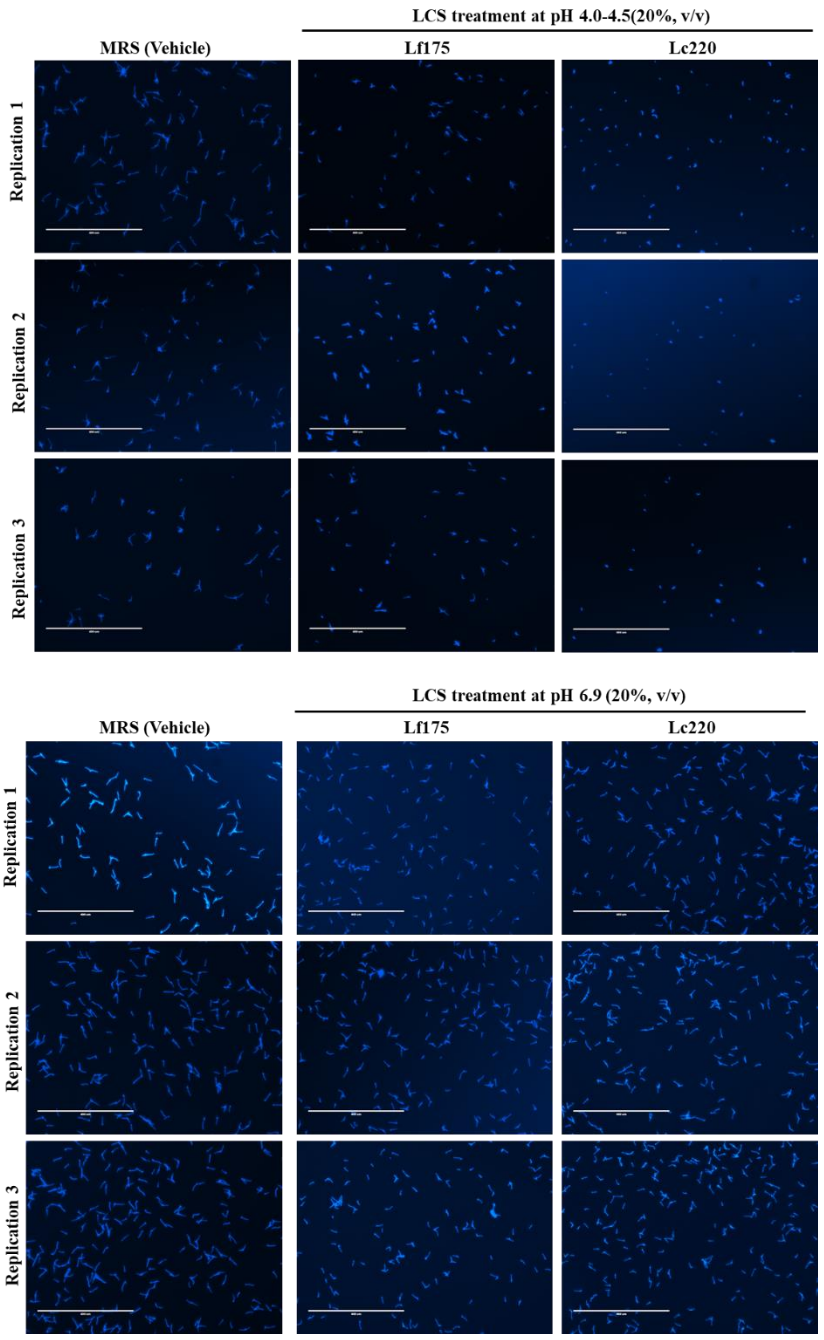


**Supplementary Figure S1. Effect of LCSs on *C. albicans* hyphal growth.** *C. albicans* was treated with LCSs at acidic (4.0–4.5) and neutral (6.9) pH. Hyphae were stained with Calcofluor white and examined under a fluorescence microscope. Scale bar: 200 μm. Lf175 and Lc220 represent *L. fermentum* SNUV175 and *L. crispatus* SNUV220, respectively.

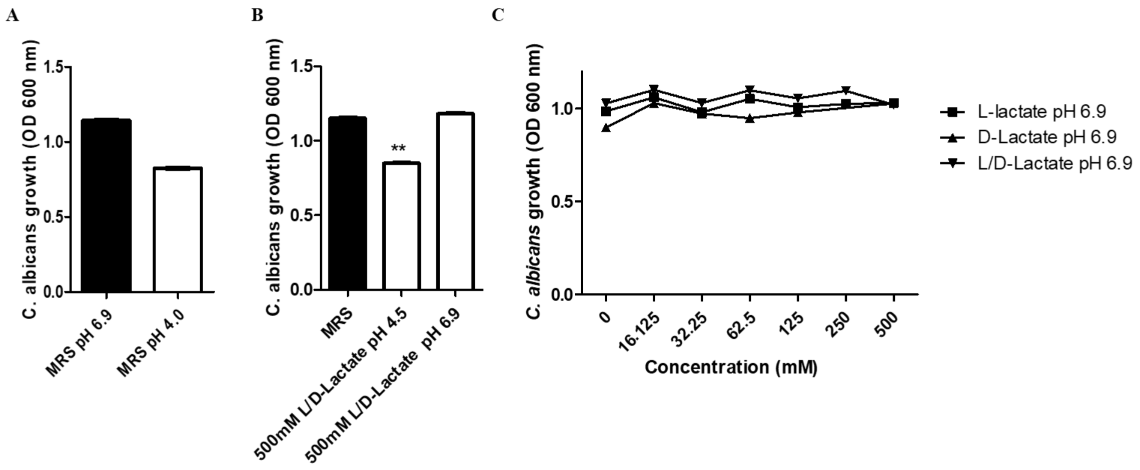


**Supplementary Figure S2. Effect of pH and lactate concentration on *C. albicans* growth.** (A–B) Effects of pH (A) and lactate (B) on *C. albicans* growth (N=2). (C) Effects of various concentrations of lactate isomers on *C. albicans* growth. Statistical significance was calculated using the Mann-Whitney U test. **, $p<0.001$.

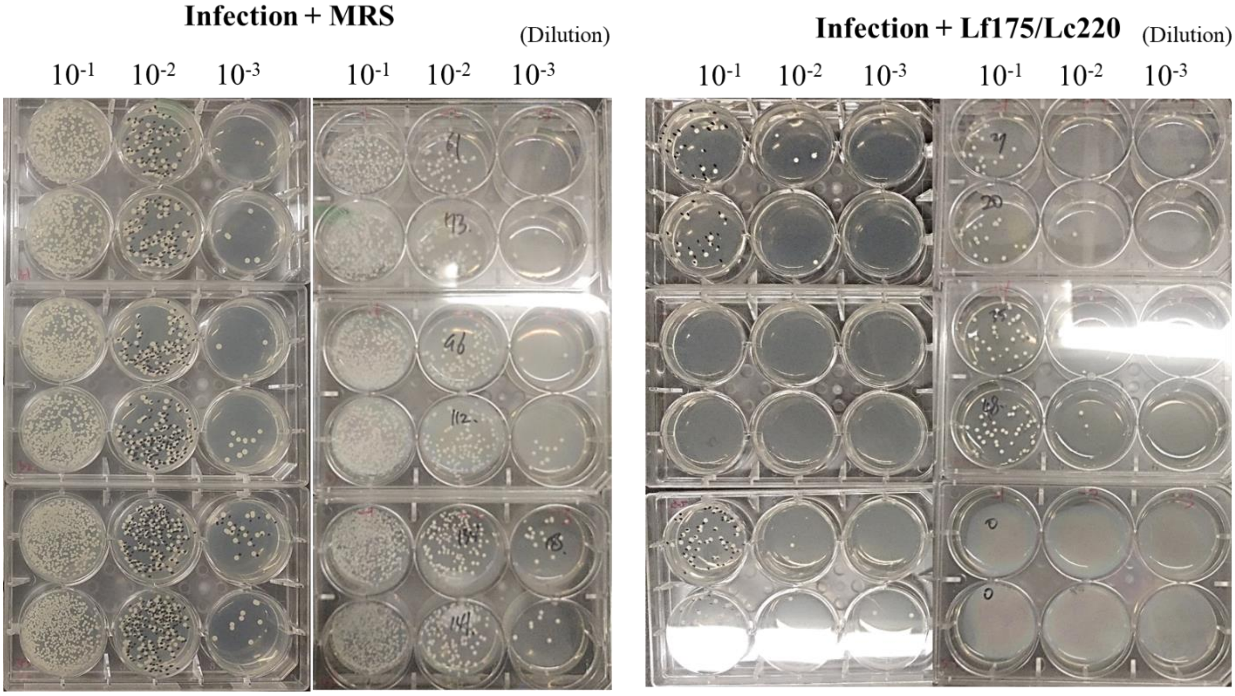


**Supplementary Figure S3. Effect of LCSs on vulvovaginal candidiasis**. Vaginal fluid was harvested from *C. albicans*-infected mice treated with MRS or a mixture of LCSs from Lf175 and Lc220. Colony forming units (CFUs) were counted. Vaginal fluid was diluted with PBS. Lf175 and Lc220 represent *L. fermentum* SNUV175 and *L. crispatus* SNUV220, respectively.
